# Tooth-Related Disease Detection System Based on Panoramic Images and Optimization Through Automation: Development Study

**DOI:** 10.2196/38640

**Published:** 2022-10-31

**Authors:** Changgyun Kim, Hogul Jeong, Wonse Park, Donghyun Kim

**Affiliations:** 1 AI Cloud R&D Center, InVisionLab Inc Seoul Republic of Korea; 2 Department of Advanced General Dentistry, College of Dentistry, Yonsei University & Institute for Innovation in Digital Healthcare Seoul Republic of Korea

**Keywords:** object detection, tooth, diagnosis, panorama, dentistry, dental health, oral health, dental caries, image analysis, artificial intelligence, detection model, machine learning, automation, diagnosis system

## Abstract

**Background:**

Early detection of tooth-related diseases in patients plays a key role in maintaining their dental health and preventing future complications. Since dentists are not overly attentive to tooth-related diseases that may be difficult to judge visually, many patients miss timely treatment. The 5 representative tooth-related diseases, that is, coronal caries or defect, proximal caries, cervical caries or abrasion, periapical radiolucency, and residual root can be detected on panoramic images. In this study, a web service was constructed for the detection of these diseases on panoramic images in real time, which helped shorten the treatment planning time and reduce the probability of misdiagnosis.

**Objective:**

This study designed a model to assess tooth-related diseases in panoramic images by using artificial intelligence in real time. This model can perform an auxiliary role in the diagnosis of tooth-related diseases by dentists and reduce the treatment planning time spent through telemedicine.

**Methods:**

For learning the 5 tooth-related diseases, 10,000 panoramic images were modeled: 4206 coronal caries or defects, 4478 proximal caries, 6920 cervical caries or abrasion, 8290 periapical radiolucencies, and 1446 residual roots. To learn the model, the fast region-based convolutional network (Fast R-CNN), residual neural network (ResNet), and inception models were used. Learning about the 5 tooth-related diseases completely did not provide accurate information on the diseases because of indistinct features present in the panoramic pictures. Therefore, 1 detection model was applied to each tooth-related disease, and the models for each of the diseases were integrated to increase accuracy.

**Results:**

The Fast R-CNN model showed the highest accuracy, with an accuracy of over 90%, in diagnosing the 5 tooth-related diseases. Thus, Fast R-CNN was selected as the final judgment model as it facilitated the real-time diagnosis of dental diseases that are difficult to judge visually from radiographs and images, thereby assisting the dentists in their treatment plans.

**Conclusions:**

The Fast R-CNN model showed the highest accuracy in the real-time diagnosis of dental diseases and can therefore play an auxiliary role in shortening the treatment planning time after the dentists diagnose the tooth-related disease. In addition, by updating the captured panoramic images of patients on the web service developed in this study, we are looking forward to increasing the accuracy of diagnosing these 5 tooth-related diseases. The dental diagnosis system in this study takes 2 minutes for diagnosing 5 diseases in 1 panoramic image. Therefore, this system plays an effective role in setting a dental treatment schedule.

## Introduction

### Usage of Medical Data and Artificial Intelligence in Health Care

Several recent studies [1-3] have used various medical data for eHealth care, but they are merely adding digital and network functions to the existing medical equipment, and remote services included in the treatment are unused. In addition, although eHealth care processes medical data and information through the networking function of doctors and patients, in reality, patients cannot obtain and confirm much information. Although a large amount of medical data has been accumulated, there has been a limit to using these data to provide information to patients and find new practical implications. As the importance of medical data has increased, a clinical data warehouse has been established to research how to utilize various medical data and for patients to find medical information easily through the provision of public and private medical data [4]. Various studies on the application of big data and artificial intelligence (AI) in medicine have shown that the University of North Carolina Healthcare has dramatically reduced the time and effort of medical staff by performing unstructured medical data analysis using content analytics and natural language processing and automatically extracting abnormal parts by machine reading and automatic processing algorithms in mammography screenings and pap smears [5]. Patients’ conditions are diagnosed remotely after the initial treatment by clinical professionals providing them with the medical information to manage their disease [6]. Recently, a method that allows users to easily use various medical data based on their experiences and help them make decisions through optimal information delivery when applying it to medical systems has been studied [7]. Using medical data and AI, patients can prevent diseases in advance and increase their autonomy in treatment scheduling by receiving knowledge of their condition and medical information. In addition, AI using medical data can reduce medical time and cost by assisting doctors in treatment.

### Dental Caries Diagnosis Using Images

Dental caries is diagnosed using videos and radiographs, and studies [8,9] have shown the processing of videos and images for a more accurate diagnosis of dental caries. In 2003, Møystad et al [10] diagnosed dental caries by using pre-enhanced Digora storage phosphor images while performing radiography on areas where tooth decay occurred and where panorama X-ray and computed tomography systems (Soredex Medical Systems) could not be used because of territorial issues. In 2017, Veena Divya et al [11] diagnosed dental caries by using the contrast map of a panoramic image, controlling the contrast of the bright and dark parts to make the blurred panoramic image clear. In the same year, Singh and Sehgal [12] added light contrast to panoramic images to enhance the clarity and diagnose dental caries by exploring the dark areas, which corresponded to dental caries in the images. In 2019, Kale et al [13] showed that mothers were able to diagnose dental caries in photos of normal and decayed teeth obtained with a smartphone by using an atlas. In 2020, the Laplacian filtering backpropagation algorithm was used to learn and diagnose dental caries [14]. In 2021, Bayraktar and Ayan [15] diagnosed dental caries by using image deep learning algorithms; that study used 1000 radiographic teeth data points for learning and validation. Labeling the dental caries was performed by a professional dentist, and dental caries in the premolars and molars were examined [15].

### Importance of Dental Caries Diagnosis

Dental caries is one of the most common chronic diseases worldwide. Oral diseases are recognized as serious diseases like other systemic diseases and were classified by the World Health Organization in 2011 as serious noncommunicable diseases. The teeth are one of the most important organs in the body, and dental caries is one of the biggest causes of tooth disease [16]. Dental caries develop and progress in 4 stages, starting as a tiny black spot in stage 1, followed by enamel decay in stage 2, nerve damage in stage 3, and pulp damage and pus and inflammation in stage 4. Dental caries can be easily repaired with simple treatment in stages 1 and 2; however, if the initial stages 1 and 2 are not judged or are overlooked, dental caries progress to stages 3 and 4. This leads to complications such as toothache, inflammation, and acute osteomyelitis, which destroys the bones around the teeth. Therefore, it is important to prevent and manage dental caries. The management and early removal of dental caries through an initial diagnosis are essential factors for good dental health [17]. However, if there are no clinical symptoms in the early stages of dental caries, people often do not pay much attention. In addition, since dental treatment is generally performed to promptly resolve uncomfortable areas, dentists can also pass over without diagnosing any of the following: proximal caries, which occurs between teeth; periapical radiolucency, which occurs from the root apex; and residual root in the bone. Therefore, to solve this problem, AI can help dentists diagnose early dental caries and other tooth-related diseases that may be difficult to judge visually by using panoramic images. Through this system, dentists and patients can reduce treatment planning time and easily treat tooth problems before they worsen, and patients can identify problems with their teeth and improve their quality of life by preventing diseases that could occur in the future.

Although various simple and easy AI diagnostic methods in the dental field have been studied, there are limitations [18] in diagnosing dental caries accurately in tooth sections. Since previous models have been used for diagnosing dental caries in the entire tooth, there are limitations in diagnosing dental caries that require precise diagnosis, such as proximal and root caries. This study aims to learn and diagnose 5 tooth-related diseases (ie, coronal caries or defects, proximal caries, cervical caries or abrasion, periapical radiolucency, and residual root) by using image deep learning models, which can assist dentists’ diagnosis by reducing treatment planning time.

## Methods

### Data Collection

Since this study evaluated 5 tooth-related diseases (ie, coronal caries or defects, proximal caries, cervical caries or abrasion, periapical radiolucency, and residual root), which are commonly diagnosed using dental imaging, training data were generated by collecting and labeling panoramic images with tooth-related diseases. This study used panoramic images provided by 50 dental clinics from 2001 to 2021. Data from 30 dental hospitals in Korea were collected, anonymized, and used for this study. Among the anonymized genders, there were 3702 males and 3783 females, with a total of 2515 unidentified persons who could not be identified. Population distribution by age group did not include teenagers; there were 1721 persons in their 20s, 956 persons in their 30s, 1134 persons in their 40s, 1351 persons in their 50s, 1914 persons in their 60s and older, and 2934 persons with unknown identities.

**Figure 1:**
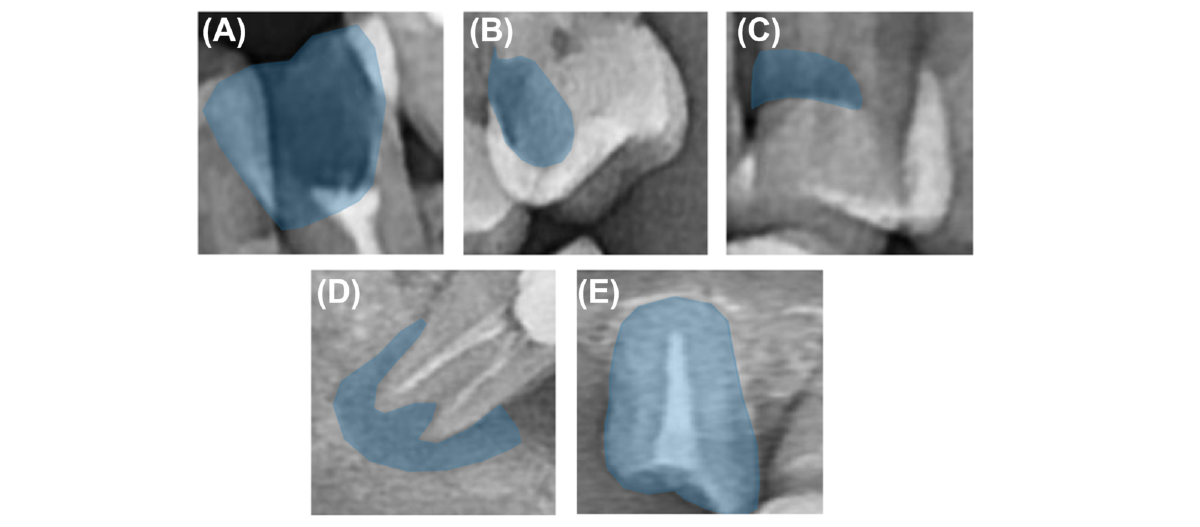
Findings of each tooth-related disease (ie, coronal caries or defect, proximal caries, cervical caries or abrasion, periapical radiolucency, residual root, in clockwise order from the top left).

A total of 10,000 panoramic images with one or more of the following 5 tooth-related diseases were used for labeling: 4206 images of coronal caries or defects, 4478 images of proximal caries, 6920 images of cervical caries or abrasion, 8290 images of periapical radiolucency, and 1446 images of residual roots. As shown in Figure 1 and Table 1, coronal caries or defects showed defects or radiolucencies that lacked density compared to the normal in the coronal portion of the tooth, proximal caries showed radiolucency that lacked density compared to the normal in the adjacent surfaces between teeth, and cervical caries or abrasion showed radiolucency that lacked density compared to the normal in the cervical area of the tooth. In addition, periapical radiolucency showed a lower density than normal radiolucency in the periapical area, and residual root means that the coronal portion is completely lost and only the root portion remains. Each label was created by focusing on these findings on the panoramic images. We used 10,000 images of male and female Koreans to label each tooth-related disease. Radiologic specialists with over 20 years of dental imaging experience performed the labeling. It took 2 minutes on average for the radiologic specialists to read the 5 diseases presented in Table 1 on 1 panoramic image of the tooth, and it took approximately 6 hours on average to read 100, including the break time. Therefore, it took approximately 50 days to label 10,000 samples. Table 1 shows the standards agreed upon by the graders. This standard is presented in Oral Radiology: Principles and Interpretation [19].

**Table 1:** Findings of each tooth-related disease.

Tooth-related diseases	Findings
Coronal caries or defect	Defect or radiolucency that lacks density compared to normal in the coronal portion of a tooth
Proximal caries	Radiolucency that lacks density compared to normal in the adjacent surfaces between teeth
Cervical caries or abrasion	Radiolucency that lacks density compared to normal in the cervical area of the tooth
Periapical radiolucency	Low density compared to normal in the periapical area of tooth
Residual root	Coronal portion of tooth is completely lost and only the root portion remains

### Learning Model (Designing and Training the Model)

Labeling was performed by data collection and preprocessing, and thus, an image classification model was used to learn about each of the 5 tooth-related diseases. This study learned dental diseases by using fast region-based convolutional network (Fast R-CNN), residual neural network (ResNet), and inception. The model with the highest accuracy in disease detection was selected. For training the model, 10,000 panoramic images were modeled in total: 4208 coronal caries or defects, 4478 proximal caries, 6920 cervical caries or abrasion, 8290 periapical radiolucency, and 1446 residual roots.

### Model Used in This Study (Additional Case of Model Application)

**Figure 2:**
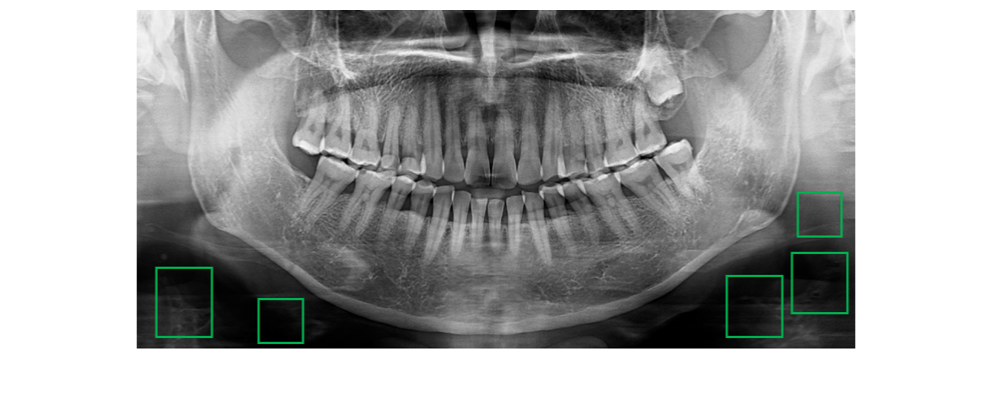
Case of misclassification of tooth-related diseases. The green boxes represent detected areas that are not included in the teeth.

Fast R-CNN has increased accuracy compared to the existing object detection algorithms because it extracts the image features and minimizes the noise in image analysis. Fast R-CNN consists of a convolution feature map and a region of interest feature vector [20]. The convolution feature map delivers the image to the convolution and max-pooling layers, and the received information is placed as a feature in the region of interest. Thereafter, the feature vector map is converted into a map with various features, and the object value of the object image of class K is determined by moving to the fully connected layers [21]. In this process, multiple work losses are minimized, and the learning accuracy is improved by using a loss function. Learning multiple classes of tooth-related diseases in 1 Fast R-CNN model sometimes results in errors in the detection of panoramic images with dark areas, as shown in Figure 2. Therefore, this study applies a single class to 1 Fast R-CNN model instead of multiple classes to improve the accuracy of detecting tooth-related diseases.

**Figure 3:**
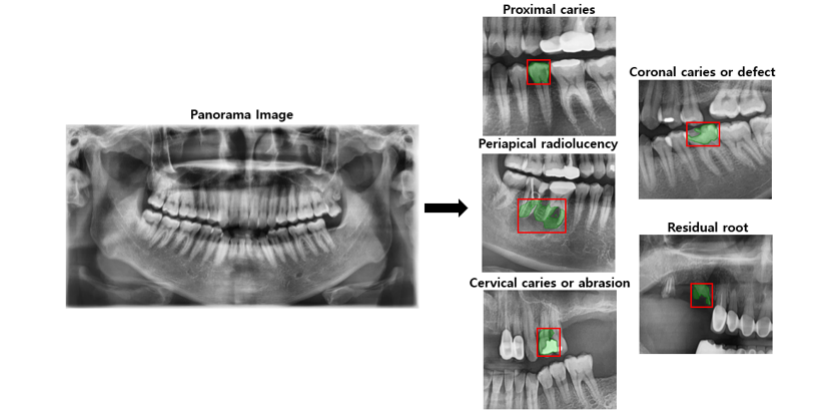
Bounding box for each tooth-related disease.

For image reading, a rectangular bounding box was first used, and segmentation was performed through an algorithm based on about 500 segmentation data. In the case of segmentation, accuracy was not calculated for the segmented data because it was used only for grasping the approximate accuracy. Thereafter, the coordinate values of the box-type tooth classes that are multilabeled in 1 tooth panoramic image were derived. Each disease corresponding to the derived coordinate value was classified by class. Then, each of the 5 tooth classes was applied to learning through the box coordinate values having the corresponding dental disease on the panoramic image. Through this, the input value for 1 model was constructed using the panoramic image data of 1 class and the box coordinate values corresponding to dental diseases. As shown in Figure 3, a bounding box was designated for each tooth-related disease, and the classes for each tooth-related disease were defined.

ResNet derives a value through the weight layer to solve the problem of overfitting owing to increased dimensional depth in deep learning, which adds the result learned through the previous weight layer to the activation function and delivers it to the next layer [22]. Therefore, this learning method, even if the depth of the learning layer deepens, solves the overfitting problem because important weights can be used for the next learning without forgetting the past learning results [23]. Because of these advantages, in this study, deep-layer learning is required to derive detailed results in learning panoramic images with similar image characteristics, and the ResNet model that can learn such a model was selected.

**Figure 4:**
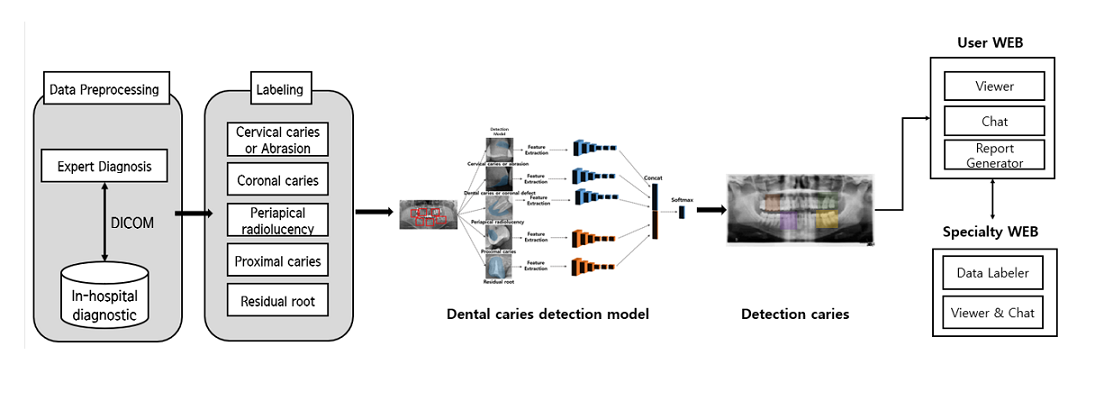
Integrated detection system for the learning process. DICOM: digital imaging and communications in medicine.

Inception, like ResNet, is created to solve the overfitting problem and the increase in computational traffic through a lot of learning when the size of the model is increased by increasing the depth of the layer [24]. In the inception model, it is possible to derive results in a fast learning time by using a small number of calculations, even in a model with a complex structure, by connecting only nodes with a high relationship between each node [25]. In addition, using various convolution filters, we derived a model that can make optimal judgments based on the features derived from each filter. This study evaluated 5 tooth-related diseases by using 3 models: Fast R-CNN, ResNet, and inception. To increase the detection accuracy for 5 tooth-related diseases, a model was designed through a process shown in Figure 4 (additional model), and the 5 tooth-related diseases were learned through Fast R-CNN, ResNet, and inception. In learning tooth-related disease data (the result of the additional model), the 3 models provided good performance for multi-class learning. However, for each part of the panoramic image composed of the contrast ratio of white and black, if multiple classes are learned in one detection model for tooth-related diseases that have similar characteristics but different sizes, there were cases where the black background was detected as a tooth-related disease. As the learning proceeded by inputting data for 5 tooth-related diseases as a whole, more black screens were learned, and the results are shown in Figure 2. As shown in the box in Figure 2, there are cases where areas such as the background of other panoramic images that are not included in the teeth are detected. To solve the problem of multi-class learning, as shown in Figure 2, professional reading experts labeled 10,000 images in a bounding box form with 5 dental diseases in a single tooth image and finally converted it into the CSV format. Label information corresponding to each dental disease was extracted from the data set containing the labeling information of 5 dental diseases, and each data set was derived for each of the 5 dental diseases. Therefore, 5 CSV-format data sets that were composed of panoramic images were modeled in total: 4208 coronal caries or defects, 4478 proximal caries, 6920 cervical caries or abrasion, 8290 periapical radiolucency, and 1446 residual roots. Further, depending on the model, DICOM (digital imaging and communications in medicine) to BMP (bitmap) conversion was performed, and auto brightness correction and adjustment were partially performed.

### Development of an Integrated Detection System for Tooth-Related Diseases

While learning about the 5 tooth-related diseases through Fast R-CNN, ResNet, and inception, problems, as shown in Figure 2, appeared. To solve these problems, a single model was applied to 1 class of tooth-related diseases to create a training model for each of the 5 tooth-related diseases so that varying locations and sizes of the diseases could be detected in detail.

**Figure 5:**
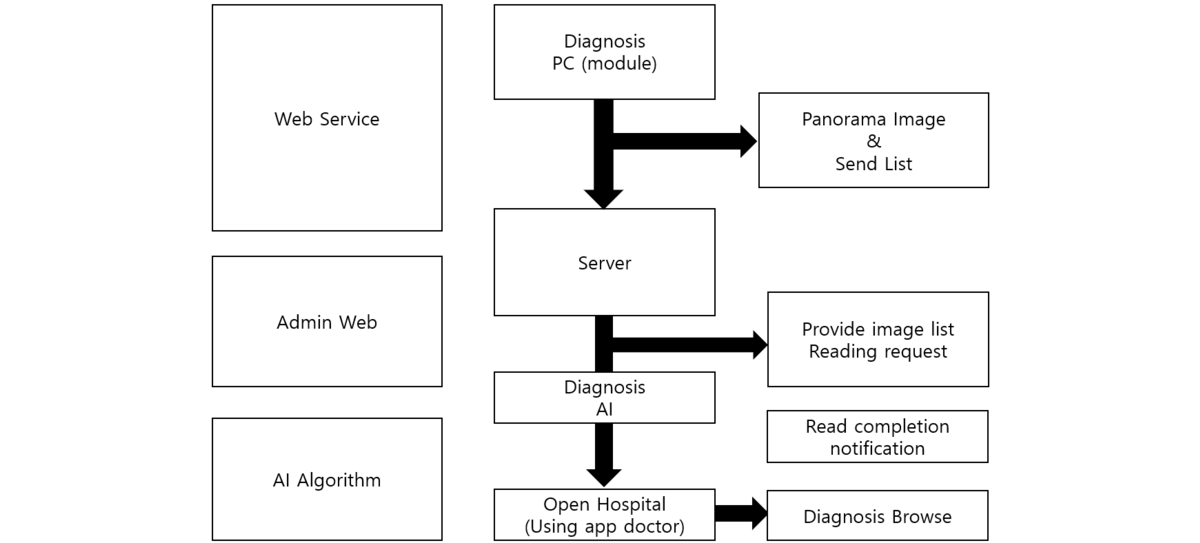
Flow diagram of the learning process. AI: artificial intelligence; PC: personal computer.

Based on the process shown in Figure 4, 5 tooth-related diseases were learned, and dentists and experts designed a real-time diagnosis, as shown in Figure 5. In designing the process, the service and administrator web were implemented using Python (version 3.6)-based Flask [26] engines (version 1.0.0), and the web page configuration was implemented using Jinja template-based HTML and Vanilla JavaScript [27]. The communication part of the AI application programming interface was composed of a Python-based Flask engine, which was installed within the Flask engine through model learning using TensorFlow 2.0.0 (Google Brain Team). Additionally, the image data of the database server were divided into file name, photographing date, patient name, patient age, image labeling prediction model data, and image labeling correct answer data to assist the dentist in the diagnosis. In the form of training/validation/test, splits were first performed and then labeled. A total of 6000 pieces were used for training, 2000 pieces were used for validation, and the remaining 2000 pieces were used for test splitting. In fact, we used ResNet/inception as the backbone of Fast R-CNN. As the input value for one model, learning data were constructed using the panoramic image data of one class and the box coordinate values corresponding to dental diseases. Through this, the input value for one model was constructed using the panoramic image data of one class and the box coordinate values corresponding to dental diseases.

In the training layer structure of each model of Fast R-CNN, ResNet, and inception, looking at the structure of the Fast R-CNN model (region proposal → CNN classification → region of interest projection), region of interest projection and bounding box regression were performed through region of interest pooling. The model is configured as shown in Figure 5, and 300 range boxes for each dental disease were specified using the CNN model in the region proposal for dental disease detection, and the features of the range corresponding to a specific class were identified. At this time, after converting features of fixed sizes in the region of interest pooling layer into a feature map, a feature vector was generated with a fully connected layer corresponding to each feature. At this time, for each feature, the position of the corresponding class was predicted using SoftMax and Bbox regressor. The epoch of model training was performed 100,000 times, and the learning rate was set to 0.001.

ResNet improves the accuracy by reducing the depth of the learning layer and increasing the performance compared to the CNN model, which is an existing image analysis model, through residual learning. In order to increase the learning accuracy in general CNNs, many layers are stacked. However, such a deep layer can lower the accuracy of the learning model. When learning through residual learning, the positive error rate can be lowered even when learning in a deep layer. When ResNet derives a value from the weight layer through the activation function in the convolution operation, it imports the previously learned information as it is, as shown in Figure 5, and learns the residual information, F(x). Looking at the formula, when the input value x is input, the first weight value is multiplied, and the activation function is multiplied by the second weight value. At this time, it is additionally multiplied by x identity, that is, x value. Therefore, since the result is derived through continuous repetition of this process, y is derived by adding a multiple convolutional layer F(x,{W_i_}) and short connection W_s_x, which takes the existing input value as it is, to the result value.


*y = F (x,{Wi}) + Wsx*

In this way, by adding information to the result derived from the weight layer, information can be added and computational complexity can be reduced so that a model with faster learning and better performance can be derived. Since ResNet learns 1 dental disease by using 5 models as 1 model, 50 hidden layers of each dental disease were designated for learning. For training, like Fast R-CNN, the training epoch was performed 100,000 times, and the learning rate was set to 0.001.

**Figure 6:**
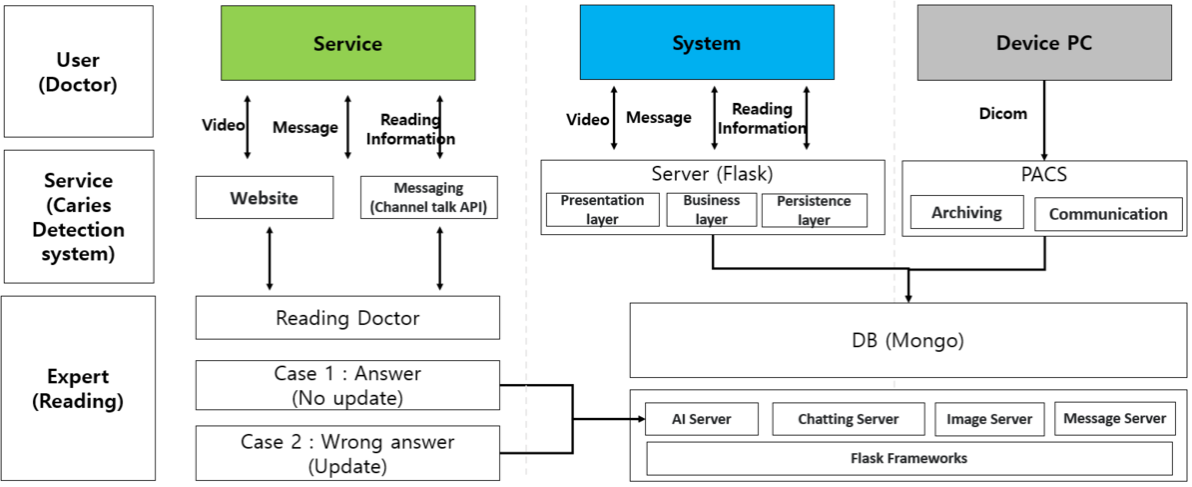
Schematic diagram of a detection system for tooth-related diseases. AI: artificial intelligence; API: application programming interface; DB: database; PACS: picture archiving and communication system; PC: personal computer.

The inception model connects the highly correlated nodes when the correlation between each node is high in the fully connected architecture and does not connect the rest so that N clusters are created for each feature. When creating a connected architecture, we additionally convolve features that are far from each other through filters of various sizes for nonuniform and inefficient sparse structures and reduce the number of channels by using a 1 × 1 filter for nodes with high correlation. The inception model was constructed, as shown in Figure 6. For the model configuration, a dental disease detection model was built using 10 pooling layers. The training epoch was performed 100,000 times, and the learning rate was set to 0.001. When a list of images is received from a computer connected to the X-ray equipment and the data are stored in the server database, a separate image is retransmitted to a system that is requested to be read from the stored data. Thereafter, it provides information read through a detection model for tooth-related diseases in real time so that it can assist dentists in shortening the reading time.

The overall flow diagram is shown in Figure 6 and is divided into service, manager, and AI algorithm categories. In the service web, the data for each tooth-related disease previously labeled by experts and the updated panoramic images are continuously accumulated and provided to the server. In the manager app, the accumulated data are transmitted to the server, and the transmitted panoramic image is read by dental experts to determine the tooth-related disease. Then, the analysis data are collected through labeling, and the collected data are used to derive the result by using an AI algorithm.

Based on the process shown in Figure 5, the detailed process of the tooth-related disease determination system proposed in this study was constructed, and it can be divided into 3 parts (service, system, and personal computer). The service part is designed to receive panoramic image data and read information through the website, and the messaging system is designed for users to communicate through the channel talk application programming interface [23]. The information provided to the readers was labeled so that the AI model could be learned, and it was designed to enable continuous data updates. In addition, when the labeling result was applied to the AI model and the AI result was judged again by the reader, it was updated to Case 1 if it was correctly judged and to Case 2 when the judgment was incorrect. Therefore, after being read accurately again by the reader, the accuracy of the model was improved through continuous data updates with the AI server. In the system, a server was built to enable the website of the service part to work. The server was built based on Flask, and it was largely divided into the presentation, business, and persistence layers [28,29]. The server connects the user and client systems through 3 layers and enables the movement of data in the database. The database was designed using MongoDB [30], which can quickly operate various types of data. AI, chatting, image, and message servers were built into MongoDB to increase the real-time movement speed of the data. The AI server, which plays a role in providing tooth-related disease reading results, updates the results of expert reading provided by doctors and provides the doctor with tooth-related disease results on new images to improve accuracy through mutual feedback, which helps users to understand by providing feedback on the opinions of users on the personal computer. Finally, it stores the dental panoramic image provided through the image server or provides medical information to personal computer users so that they can view and continuously manage the medical records whenever necessary.

### Ethical Considerations

Since the data is a retrospective study, it was processed in the direction of protecting personal information through database anonymization, etc. In addition, data collected for research purposes were collected through Cheongju University Bioethics Committee IRB (1041107-202208-HR-024-01).

## Results

### Detection System

**Figure 7:**
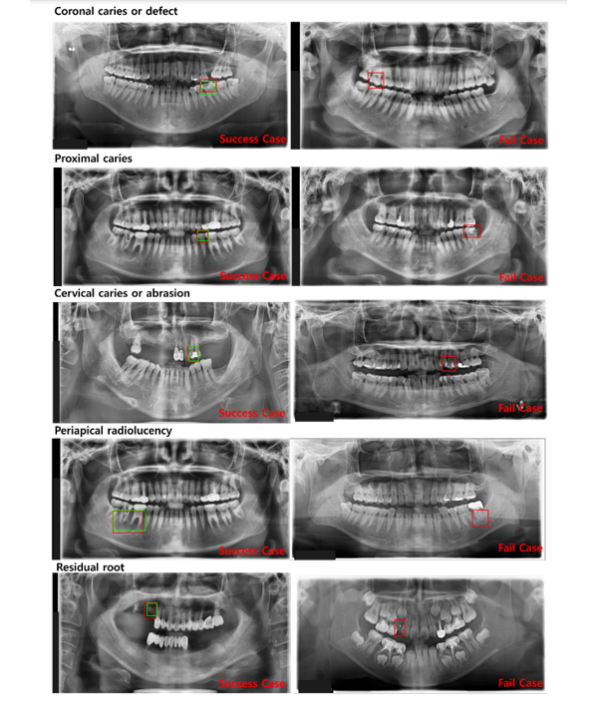
Success and fail cases in the detection of the 5 dental diseases.

**Figure 8:**
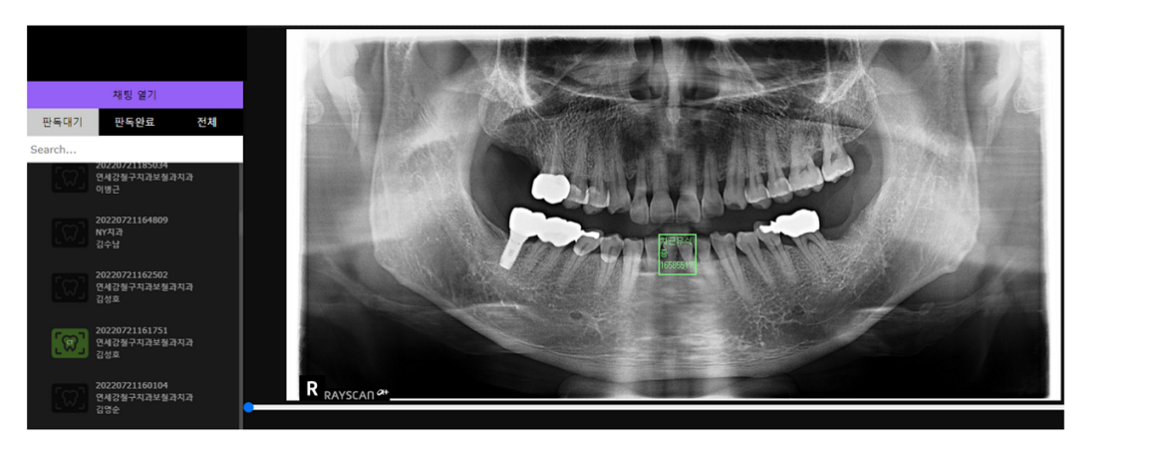
Web system for tooth-related diseases.

The web service in this study was built based on the process presented in Figure 6 and Figure 7. As shown in Figure 7, panoramic images facilitate faster judgment of dental-related diseases than the conventional doctor’s diagnosis techniques. The detection system aids and shortens the treatment time through the transmission of images taken in real time. Figure 7 shows a case in which a tooth disease was correctly judged and another case in which a dental disease was incorrectly judged. Since cases can be judged inaccurately, doctors can use this auxiliary system to check the patient’s condition once again. Figure 8 shows that patients can check their panoramic images on the web, and they can know about the treatment plan and receive information on tooth-related diseases for effective disease management.

### Benefits of Using Web-Based Systems and User Interface

To detect tooth-related diseases, Fast R-CNN, which has the best performance among the classification models from the point of view of a dentist, was applied. For learning the model for judging the 5 types of tooth-related diseases, dentists can update the panoramic images in real time through the web application programming interface and continuously collect data by improving the accuracy through additional updates on the tooth-related disease detection labeling. Figure 6 shows how the tooth-related disease judgment web service screen appears. Figure 6 shows that by providing doctors and patients with the diagnosis of their diseases through the patient’s panoramic image, past medical records, and current status on the web, doctors can provide prompt treatment for dental diseases and patients can monitor their dental status. Therefore, from the patient’s perspective, users can check medical records and treatment areas through the web screen of the panoramic image provided by the hospital where they have been treated and check for tooth-related diseases. In addition, because the treatment time and the subsequent treatment times can be known, users can use this system to manage their tooth-related diseases, which require continuous management.

### Model Comparison Results

This study created a detection model for 5 dental diseases that are difficult to judge visually (ie, coronal caries or defect, proximal caries, cervical caries or abrasion, periapical radiolucency, and residual root) by using a dental panoramic image. Fast R-CNN, ResNet, and inception have previously been used to learn about dental disease detection [20,22,31]. In training the model, 4206 cases of coronal caries or defects, 4478 cases of proximal caries, 6920 cases of cervical caries or abrasion, and 8290 cases of periapical radiolucency, and 1446 cases of residual roots were trained among a total of 10,000 panoramic images. Therefore, a model for judging the 5 types of dental caries using 1 panoramic image was developed by creating a training model for each dental disease into one detection model through an integrated detection system for dental diseases. Regarding the number of training sessions, all 3 models were trained 200,000 times, the results were compared, and the model with the highest accuracy was selected. The results of deriving the precision, sensitivity, and specificity of the detection results for the 5 dental diseases are shown in Figure 8. As shown in Figure 8, the coronal defect showed the highest specificity, with an average specificity of 90 or more. In addition, the sensitivity was found to be above 80 on average, indicating that it would show high accuracy even when other data were used for learning.

Table 2 shows the results of learning with Fast R-CNN, ResNet, and inception for the 5 tooth-related diseases. As shown in Table 2, 5 tooth-related diseases were detected with an average accuracy of over 90%. Also, as shown in Figure 6, the specificity is the highest for the 5 tooth-related diseases. This means that each tooth-related disease can be detected with high accuracy. With the tooth-related disease detection web service presented in this study, considerable time can be saved in diagnosing tooth-related diseases. On average, it takes about 1 minute for dental doctors to judge 5 dental diseases on 1 panoramic image. However, if the system proposed in this study is used, the results of the classification model can be judged at once through the user interface, and the time can be reduced to about 10 seconds in judging dental diseases. Therefore, it is judged to be an effective system to assist in the judgment of dental diseases.

**Table 2:** Tooth-related disease detection results.

Model, diseases		Precision	Sensitivity	Specificity
**Fast region-based convolutional network**				
	Coronal caries or defect	0.785	0.708	0.982
	Proximal caries	0.484	0.792	0.918
	Cervical caries or abrasion	0.795	0.767	0.952
	Periapical radiolucency	0.824	0.953	0.895
	Residual root	0.640	0.904	0.972
**Inception**				
	Coronal caries or defect	0.253	0.609	0.848
	Proximal caries	0.327	0.783	0.883
	Cervical caries or abrasion	0.444	0.707	0.785
	Periapical radiolucency	0.371	0.946	0.556
	Residual root	0.232	0.893	0.873
**Residual neural network**				
	Coronal caries or defect	0.2101	0.395	0.876
	Proximal caries	0.685	0.377	0.987
	Cervical caries or abrasion	0.378	0.011	0.996
	Periapical radiolucency	0.308	0.883	0.451
	Residual root	0.225	0.744	0.89

## Discussion

### Strengths and Limitations

This study has several advantages. The use of panoramic images of individual patients in dentistry is a complex procedure. This study designed a model that could determine 5 types of dental caries by acquiring various panoramic image data and collecting 10,000 pieces of data with various oral structures and dental caries. Therefore, a tooth-related disease determination system with high accuracy and without complex procedures was developed. However, since there is a large deviation in the number of classes for each tooth-related disease, there was a problem in that the learning accuracy was slightly lowered where the number of analysis groups was small. The accuracy of the model is expected to be improved by collecting and supplementing data through continuous updates by using real-time panoramic images uploaded to the web.

### Conclusions

In this study, the tooth-related disease judgment system identified 5 types of tooth-related diseases that are difficult to determine clinically (visually) by using an AI model, and this information was provided on the web to create a system that allows doctors and patients to make real-time judgments. The trained model labeled 5 dental caries through 10,000 panoramic images. Accuracy was compared using Fast R-CNN, ResNet, and inception models, which are good models for detection. Among these models, Fast R-CNN was finally used, which has the highest accuracy. Therefore, Fast R-CNN can be used to shorten the time required for the diagnosis and treatment of dental caries. In addition, by updating the captured panoramic images of patients on the web service developed in this study, the system can acquire new data and further increase the accuracy of diagnosing tooth-related diseases. Additionally, the patient can be aware of the tooth areas where he or she has received treatment, the treatment time, and the type of caries, so that he or she can adjust the schedule for the future dental visit, which will aid in continuous management of dental health. Thus, this study is meaningful as it collects learning data from cases embodied as actual services and implements a prototype-type service based on the collected data. In the future, it will be possible to develop a model for predicting overall oral diseases with panoramic images through additional learning of various dental diseases.

## Abbreviations

AI: artificial intelligence
Fast R-CNN: fast region-based convolutional network
ResNet: residual neural network
